# Computed tomography porosity and spherical indentation for determining cortical bone millimetre-scale mechanical properties

**DOI:** 10.1038/s41598-019-43686-6

**Published:** 2019-05-15

**Authors:** Oliver R. Boughton, Shaocheng Ma, Xiran Cai, Liye Yan, Laura Peralta, Pascal Laugier, James Marrow, Finn Giuliani, Ulrich Hansen, Richard L. Abel, Quentin Grimal, Justin P. Cobb

**Affiliations:** 1 The MSk Lab, Department of Surgery and Cancer, Imperial College London, London, United Kingdom; 2 The Biomechanics Group, Department of Mechanical Engineering, Imperial College London, London, United Kingdom; 3 Sorbonne Université, INSERM, CNRS, Laboratoire d’Imagerie Biomédicale, F-75006 Paris, France; 4 Department of Materials, University of Oxford, Oxford, United Kingdom; 5 Centre for Advanced Structural Ceramics, Department of Materials, Imperial College London, London, United Kingdom

**Keywords:** Translational research, Characterization and analytical techniques

## Abstract

The cortex of the femoral neck is a key structural element of the human body, yet there is not a reliable metric for predicting the mechanical properties of the bone in this critical region. This study explored the use of a range of non-destructive metrics to measure femoral neck cortical bone stiffness at the millimetre length scale. A range of testing methods and imaging techniques were assessed for their ability to measure or predict the mechanical properties of cortical bone samples obtained from the femoral neck of hip replacement patients. Techniques that can potentially be applied *in vivo* to measure bone stiffness, including computed tomography (CT), bulk wave ultrasound (BWUS) and indentation, were compared against *in vitro* techniques, including compression testing, density measurements and resonant ultrasound spectroscopy. Porosity, as measured by micro-CT, correlated with femoral neck cortical bone’s elastic modulus and ultimate compressive strength at the millimetre length scale. Large-tip spherical indentation also correlated with bone mechanical properties at this length scale but to a lesser extent. As the elastic mechanical properties of cortical bone correlated with porosity, we would recommend further development of technologies that can safely measure cortical porosity *in vivo*.

## Introduction

The mechanical properties of bone strongly influence the likelihood of a person sustaining a fracture and obtaining a good result from orthopaedic surgery^1^. Patients with lower bone mineral density, as measured by dual x-ray absorptiometry (DXA), have a higher chance of sustaining hip fractures^2^, having cementless knee replacements subside^3^ and having cemented hip replacements loosen over time^4^. Using quantitative computed tomography (QCT), QCT bone mineral density measurements can also predict the chance of fixation failing in proximal humeral fractures^5^ and rotator cuff tears^6^, as well as whether fixation of a hip fracture will remain stable over time^7^. However, there is still a need to develop tools that can reliably provide bone quality metrics for helping inform surgeons as to what surgical technique or type of implant they should use^8^. Some surgeons palpate the bone directly to assess its quality. Using this “haptic assessment” of bone quality, a study showed that there was large variation between surgeons as to what was deemed “adequate bone quality”^9^. From the imaging modalities currently available to clinicians, DXA is a useful tool and, as stated above, low BMD on DXA scans is associated with aseptic loosening of hip replacements and implant subsidence^3,4^. However, the spatial resolution of DXA is relatively low and most patients suffering from a fracture have a BMD that is not within the osteoporotic range (T score less than −2.5) on DXA^10^. There are also some patients on long-term steroid use who have normal BMD values on DXA, but increased fracture risk, and patients with osteopetrosis with increased BMD values on DXA but higher fracture risk^11^. A survey of hip surgeons found that 100% of the surgeons would use a cemented implant in patients with known osteoporosis. However, only 4% of the surgeons surveyed routinely asked for DXA scans for their patients pre-operatively^12^. This could be due to surgeons not entirely trusting the findings from the DXA scans. Quantitative CT (QCT) and peripheral high resolution peripheral quantitative CT (HR-pQCT) fairly accurately predict whole bone mechanical properties but do not yet have the spatial resolution to determine cortical porosity^13^ and, therefore, are not able to detail the main determinant of the bone’s mechanical properties at the millimetre scale^14^.

Cortical bone quality is becoming increasingly recognised as an important predictor of a person’s likelihood of fracture^15,16^. The femoral neck is arguably the most important fracture site in the human body, with 1 in 10 patients dying within one month following these fractures^17,18^. In young patients the trabecular and cortical bone fairly equally contribute to femoral neck strength. However, as patients age and the bone quality deteriorates, the trabecular bone contribution to femoral neck strength decreases and in patients with low bone mineral density (BMD) on dual x-ray absorptiometry (DXA) imaging the cortical bone contributes to 3.7 times more of the femoral neck strength than the trabecular bone^19^. Therefore, we chose to investigate the mechanical properties of femoral neck cortical bone, assessing the relationship between the bone’s porosity, as measured by x-ray computed tomography (CT), with its millimetre-scale mechanical properties.

To measure the mechanical properties of bone without extracting a sample, imaging techniques including dual x-ray absorptiometry (DXA), x-ray computed tomography (CT) and ultrasound techniques can be used, or mechanical testing techniques that do not involve extracting a sample can be employed^20^. The mechanical properties of bone are often collectively referred to as the bone quality, a combination of its material composition and structure^21^. Various indentation techniques have been investigated as potential tools for measuring the mechanical properties of bone *in vivo*, without having to extract a bone sample^22^. Indentation techniques can calculate the mechanical properties of bone at the micro-scale^23,24^ in carefully prepared samples but, until now, indentation techniques on their own have not reliably predicted millimetre-scale bone mechanical properties^22,25–27^.

When performing indentation on bone, two different techniques are commonly used: Reference point indentation and depth sensing indentation. Descriptions of the different techniques are detailed in Arnold *et al*.^22^. Briefly, reference point indentation (RPI) involves repeated indentations at the same location and the indentation distance increase (IDI) is measured. This involves the bone tissue sustaining plastic deformation and is a potential surrogate measure for the fracture toughness of bone^26^. A handheld RPI device, the Osteoprobe (Active Life Scientific, USA), has been developed but this only currently measures the “Bone Material Strength Index” and does not provide any measure of the bone stiffness^28,29^. The Osteoprobe works differently from the benchtop RPI device, the BioDent (Active Life Scientific, USA). The Osteoprobe has a peak impact force of 30 Newtons, compared to 10 Newtons with the BioDent, and a faster loading rate, compared to the cyclic loading of the Biodent^26,29^. Although, the BioDent can be used to estimate the stiffness of the bone from the unloading slope of the load-displacement curve a previous study demonstrated that the BioDent could not explain the variation in elastic modulus of bone at the millimetre scale (determined by three point bending)^26^. The BioDent uses a sharp, cono-spherical tip^26^. Spherical indentation tips have the advantage over sharp indenter tips of minimising plastic deformation^30,31^. By using a spherical tip it would be possible to indent a larger area of bone, whilst minimising plastic deformation^32^. Depth-sensing microindentation can estimate the stiffness of bone from the unloading slope of the load-displacement curve during indentation^33^ and a variety of sizes and shapes of indenter tips can be used, which is why this method of indentation was used over RPI in this study.

When discussing bone quality, fracture toughness and strength are often considered first. Bone stiffness, the ability of the bone to resist deformation^34^, is also important, particularly in the field of cementless joint replacement surgery; Bone stiffness plays an important role both in the initial press-fit insertion of a cementless implant^25,35^, and in long-term bone remodelling around the implant^36,37^. To determine the safe impaction force range during cementless hip replacement the bone stiffness must be known to calculate the “safe elastic range” of bone deformation during insertion of an implant^25^. Too little strain in the bone around the implant will lead to a reduced “elastic grip”^35^, potentially leading to implant loosening, and too much strain can lead to fracture^38^. Bone stiffness, implant stiffness and implant impaction force all determine this safe elastic range^25,39^. In addition, it has been demonstrated that bone ingrowth into a bone scaffold can be improved by first determining the local bone stiffness of the site the scaffold will be inserted and then designing a scaffold with stiffness properties closely representing the local bone stiffness^40,41^. In this study, we specifically assess bone stiffness at the millimetre length scale, referred to as the apparent elastic modulus^26,42,43^.

Our group previously assessed spherical indentation by comparing it against compression testing. Wide variability in bone stiffness, as measured by micro-indentation, was found in the porous cortical bone samples. This variation in stiffness measured by indentation led to indentation values not correlating with compression testing values^25^. We hypothesised that a larger indenter tip would be less prone to the variability of conventional micro-indentation as the larger contact area would include some of the porosity, leading to indentation results that closer correlate with compression testing.

This study, therefore, aimed to address two questions:

1. Can CT-measured porosity accurately predict femoral neck cortical stiffness and strength?
2. Can large-tip spherical indentation predict cortical bone mechanical properties at the millimetre length scale?

To answer these questions, we compared micro-CT-measured porosity and large-tip spherical indentation with compression testing, for measuring the bone stiffness and strength, ultrasound studies, for measuring the stiffness, and ashing for determining the bone mineral content. Ultrasound measurements were performed in addition to compression testing as it is unclear from the literature which is more accurate for determining the stiffness of small samples of bone. Compression testing can underestimate stiffness^44^ and ultrasound methods may overestimate it^45^.

## Results

The mass density measurements ranged from 1.4 to 2.0 mg/mm^3^, with a median density of 1.8 mg/mm^3^, similar to the reported range 1.7 to 2.0 mg/mm^3^ from a tibial cortical bone study in the literature^46^ (Table 1). CT Porosity (CTP) ranged from 2.4% to 40% with a median porosity of 9.3%. This is in the range of cortical porosity measurements reported in the literature (1.6 to 46%)^15,16,47^. The Resonant Ultrasound Spectroscopy (RUS) E3 moduli ranged from 10.4 to 22.8 GPa, with a median of 18.6 GPa, similar to the value from RUS in mid-shaft femoral cortical bone of 20.0 GPa in the axis 3 direction, reported by Bernard *et al*.^48^. Bulk-Wave Ultrasound (BWUS) C_33_ values ranged from 24.8 to 33.8 GPa with a median of 30.5 GPa. This is within the upper range of ultrasound measures of cortical bone stiffness from the literature (from tibial and mid-shaft femoral cortical bone), which vary from 10.6 to 36 GPa^46,49^. The individual BWUS results are in Supplementary Materials 1.

**Table 1:** Summary of Results: The mean, median, standard deviation and range are shown for the different measurements made on the 20 bone samples from the 20 patients.

Measurement	Mean	Median	Standard Deviation	Range
Density (mg/mm^3^)	1.8	1.8	0.12	1.4 to 2.0
CT Porosity (%)	11.2	9.3	8.4	2.4 to 40.0
RUS E3 Modulus (GPa)	17.7	18.6	3.1	10.4 to 22.8
Bulk Wave US C_33_ (GPa)	30.1	30.5	2.2	24.8 to 33.8
Indentation Modulus (GPa)	4.6	4.7	1.1	1.3 to 8.0
Compression Modulus (GPa)	9.6	9.7	2.9	2.3 to 13.9
Ultimate Compressive Strength (MPa)	125.6	128.0	26.2	60.0 to 180.0
Bone Mineral Density by Ashing (mg/mm^3^)	1.0	1.0	0.12	0.7 to 1.2

Note *n* = 20 for all measurements except for the resonant ultrasound spectroscopy (RUS) measurements where one sample could not be measured (*n* = 19 for RUS results). Note, the full data set can be viewed in Supplementary Materials 3.

**Figure 1:**
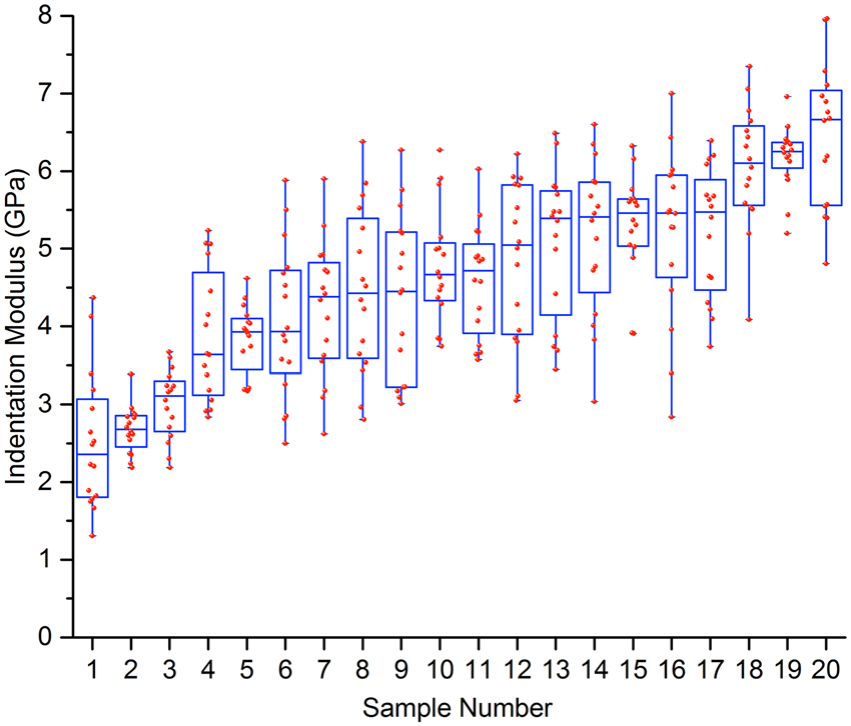
Box plot with superimposed dot plot showing the elastic moduli measured from indentation for each sample. The sample numbers on the x-axis are ordered by the median indentation modulus for each sample, from the smallest to the largest median sample indentation modulus.

The indentation moduli varied from 1.3 to 8.0 GPa, with a median of 4.7 GPa. These are lower than values in the literature for spherical-tip indentation of human femoral neck cortical bone which range from 5.8 to 13.8 GPa^25^. Figure 1 displays the variation not only between patient samples but also within the same samples. The full indentation results can be seen in Supplementary Materials 2. The compression moduli varied from 2.3 to 13.9 GPa, with a median of 9.7 GPa. This compares to reported cortical bone compression in the literature from 1.7 to 20 GPa^25,50^. The ultimate compressive strength of the samples ranged from 60 to 180 MPa, with a median of 128 MPa. This compares to a range of 100 to 191 MPa for diaphyseal femoral cortical bone^51,52^ in the literature. The bone mineral density by ashing ranged from 0.7 to 1.2 mg/mm^3^, with a median ash density of 1.0 mg/mm^3^. This compares to reported values in the literature of 0.9 to 1.3 mg/mm^3^ for diaphyseal femoral cortical bone^53^.

**Figure 2:**
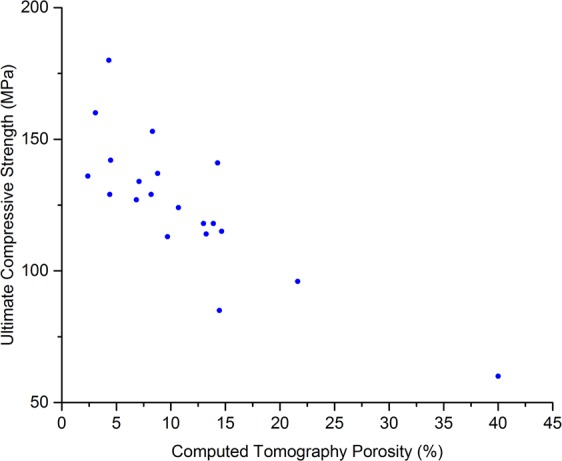
Scatter plot comparing computed tomography porosity and the ultimate compressive strength. The Spearman’s Rank Correlation Coefficient (*r*_*s*_) was −0.75, *p* < 0.001.

**Figure 3:**
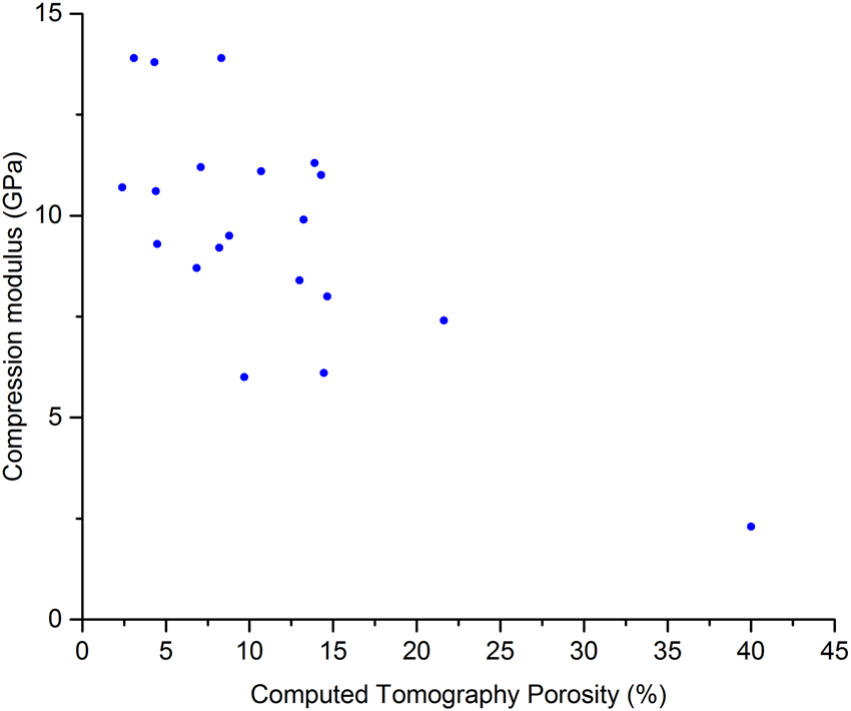
Scatter plot comparing computed tomography porosity and the apparent elastic modulus measured by compression testing. The Spearman’s Rank Correlation Coefficient (*r*_*s*_) was −0.55, *p* = 0.01.

**Figure 4:**
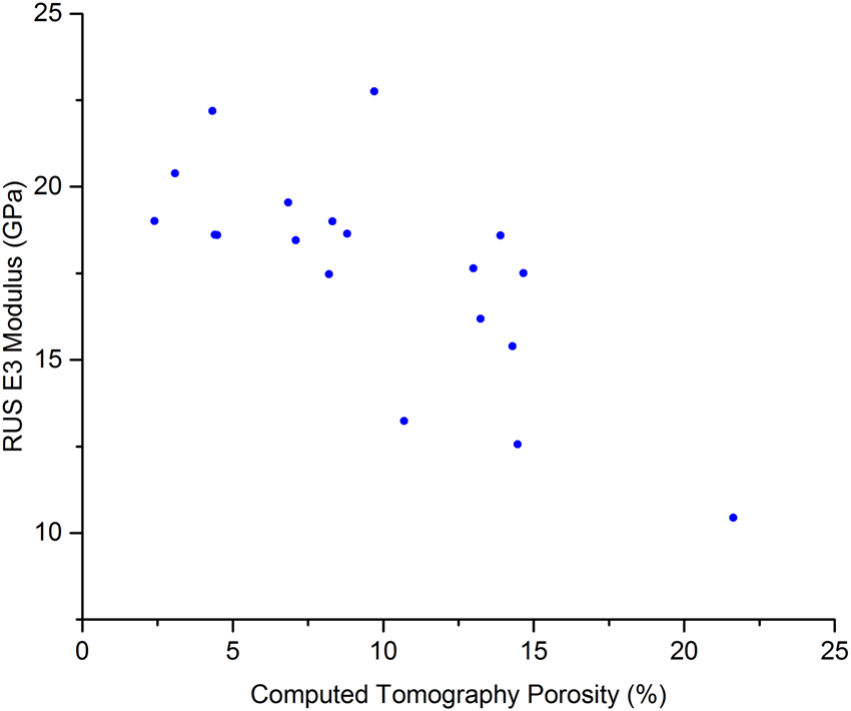
Scatter plot comparing computed tomography porosity and the modulus in the longitudinal direction (E3) measured by Resonant Ultrasound Spectroscopy (RUS). The Spearman’s Rank Correlation Coefficient, (*r*_*s*_) was −0.74, *p* < 0.001.

**Figure 5:**
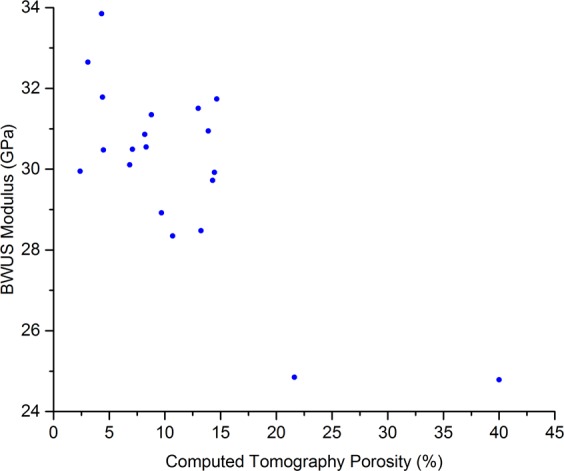
Scatter plot comparing computed tomography (CT) porosity and the stiffness in the longitudinal direction (C_33_) measured by Bulk Wave Ultrasound (BWUS). The Spearman’s Rank Correlation Coefficient (*r*_*s*_) was −0.51, *p* = 0.02.

The data were not normally distributed due to one sample having a much higher porosity than the others (40% porosity, relative to the second most porous sample having a porosity of 21.6%). Therefore, Spearman Rank Correlation Coefficients (*r*_*s*_) are reported in Table 2. The correlation between the CT porosity (CTP) and the UCS was *r*_*s*_ = −0.75 (*p* < 0.001, Fig. 2) and the correlation CTP and compression testing moduli was *r*_*s*_ = −0.55 (*p* = 0.01, Fig. 3). When comparing CTP to ultrasound measurements, the correlation between CTP and RUS measurements was *r*_*s*_ = −0.74 (*p* < 0.001, Fig. 4), and the correlation between CTP and BWUS measurements was *r*_*s*_ = −0.51 (*p* = 0.02, Fig. 5). In addition, CT porosity correlated with mass density measurements and ash density (*r*_*s*_ = −0.93, *p* < 0.001 (Fig. S1, Supplementary Materials 4) and *r*_*s*_ = −0.87, *p* < 0.001, respectively).

**Table 2:** Spearman’s Rank Correlation Coefficient values (*r*_*s*_) between different measurements. Note n = 20 for all measurements except for the resonant ultrasound spectroscopy (RUS) measurements where one sample could not be measured (n = 19 for RUS results).

	Density	CT Porosity	RUS E3 Modulus	Bulk Wave US C_33_	Indentation Modulus	Compression Modulus	Ultimate Compressive Strength	Bone Mineral Density Ashing
Density								
CT Porosity	−0.93**							
RUS E3 Modulus	0.75**	−0.74**						
Bulk Wave US C_33_	0.62**	−0.51*	0.48*					
Indentation Modulus	0.44	−0.56*	0.25	0.53*				
Compression Modulus	0.46*	−0.55*	0.29	0.44	0.48*			
Ultimate Compressive Strength	0.72**	−0.75**	0.52*	0.57**	0.56*	0.77**		
Bone Mineral Density Ashing	0.95**	−0.87**	0.62**	0.65**	0.45*	0.33	0.63**	

*Indicates p < 0.05, **indicates p < 0.01. CT = computed tomography; RUS E3 Modulus = the apparent elastic modulus measured by resonant ultrasound spectroscopy in the longitudinal (3) direction; Bulk Wave US E33 = the apparent elastic modulus in the longitudinal direction measured by bulk-wave ultrasound.

One sample had a high porosity of 40% and a low density (1.4 mg/mm^3^). The CT scan of this sample revealed that some of the bone in the sample was more porous and the sample may have been from the transition between cortical and trabecular bone. The RUS model assumes that the sample is a homogeneous material at the millimetre scale. Therefore, RUS measurements were not performed for this sample. With this single sample excluded, parametric methods of data analysis could be undertaken (least-squares regression analysis). These analyses revealed a negative correlation between CTP and BWUS (*r* = −0.70, p < 0.01, Table 3).

**Table 3:** Least-squares regression analysis, with one sample removed from the analyses due to it having a notably greater porosity (40%). The Pearson’s Correlation Coefficient (r) for each measurement comparison is shown (n = 19).

	Density	CT Porosity	RUS E3 Modulus	Bulk Wave US E33	Indentation Modulus	Compression Modulus	Ultimate Compressive Strength	Bone Mineral Density Ashing
Density								
CT Porosity	−0.91**							
RUS E3 Modulus	0.74**	−0.72**						
Bulk Wave US E33	0.78**	−0.70**	0.79**					
Indentation Modulus	0.28	−0.32	0.06	0.19				
Compression Modulus	0.48*	−0.57*	0.35	0.47*	0.41			
Ultimate Compressive Strength	0.68**	−0.75**	0.64**	0.66**	0.35	0.84**		
Bone Mineral Density Ashing	0.87**	−0.74**	0.57*	0.61**	0.17	0.27	0.47*	

*Indicates p < 0.05, **indicates p < 0.01. CT = computed tomography; RUS E3 Modulus = the apparent elastic modulus measured by resonant ultrasound spectroscopy in the longitudinal (3) direction; Bulk Wave US E33 = the apparent elastic modulus in the longitudinal direction measured by bulk-wave ultrasound.

**Figure 6:**
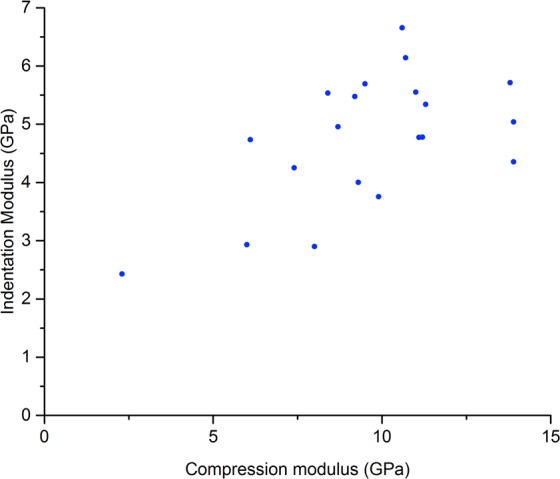
Scatter plot comparing the indentation modulus values with the compression testing apparent elastic moduli. The Spearman’s Rank Correlation Coefficient, *r*_*s*_, was 0.56, *p* = 0.01.

**Figure 7:**
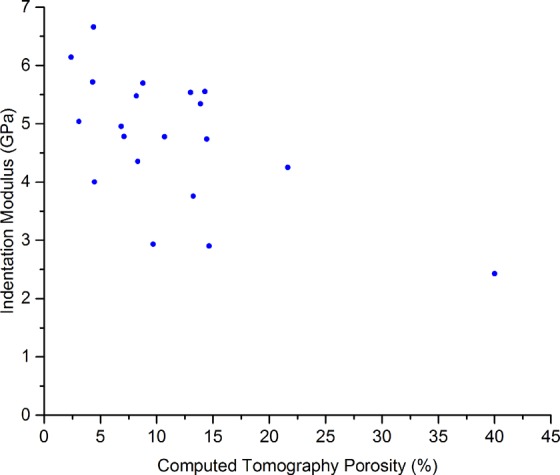
Scatter plot comparing computed tomography (CT) porosity and the indentation modulus results. The Spearman’s Rank Correlation Coefficient, *r*_*s*_, was −0.56, *p* = 0.01.

There were correlations between the spherical indentation measurements of moduli and the compression testing moduli (*r*_*s*_ = 0.48, *p* = 0.03, Fig. 6) and between the indentation moduli and UCS measurements (*r*_*s*_ = 0.56, *p* = 0.01, Fig. S2, Supplementary Materials 4). In addition, there were correlations between the indentation moduli and ash density measurements (*r*_*s*_ = 0.45, *p* = 0.049) and the indentation moduli and BWUS measurements (*r*_*s*_ = 0.53, *p* = 0.02). No significant correlations were observed between indentation moduli and resonant ultrasound spectroscopy moduli (*r*_*s*_ = 0.25, *p* = 0.3) and indentation moduli and mass density (*r*_*s*_ = 0.44, *p* = 0.05). Figure 7 shows the correlation between CT porosity and indentation measurements (*r*_*s*_ = −0.56, *p* = 0.01). When the higher porosity sample was excluded, the trends between indentation and other measurements failed to reach significance.

To assess if CT porosity combined with indentation improved the prediction of millimetre-scale mechanical properties multiple linear regression analyses were performed, with the high porosity sample excluded due to multiple linear regression analysis requiring normally distributed data (the data set for the regression analyses were 19 samples). The dependent variable was the ultimate compressive strength. The other measurements were inputted as independent variables (predictors). When CT porosity and ash density were used as independent variables, with the ultimate compressive strength as the dependent variable, the correlation was *r* = −0.76 (*p* = 0.001). This correlation was not substantially different to the correlation between CT porosity alone and the ultimate compressive strength (*r* = −0.75). CT porosity was a significant predictor (*p* = 0.002), whereas ash density was not (*p* = 0.46). When inputting CT porosity, ash density, indentation moduli and RUS data as independent variables, the correlation with ultimate compressive strength as the dependent variable was *r* = −0.79, (*p* = 0.006). This was also not substantially changed from CT porosity alone (*r* = −0.75). The *p* values for the independent predictors in this multiple linear regression were 0.06 for CT porosity, 0.47 for ash density, 0.39 for indentation moduli, and 0.30 for RUS data. As the *p* values for this multiple linear regression were all not significant the sample size of 19 was likely not sufficient to perform this analysis.

## Discussion

There were two important findings from this study. Firstly, porosity, as measured by micro-CT, was found to correlate with the stiffness of femoral neck cortical bone samples, measured by RUS (*r*_*s*_ = −0.74, *p* < 0.001), and correlate with the bone strength, measured by compression testing (*r*_*s*_ = −0.75, *p* < 0.001). To our knowledge, this is the first study that has compared CT porosity against the mechanical properties of cortical bone from the femoral neck, obtained by both compression testing and ultrasound techniques. Secondly, spherical indentation modulus values were found to correlate with the compression testing millimetre-scale mechanical properties (*r*_*s*_ = 0.48, *p* = 0.03). Previous studies that have combined indentation with CT imaging have predicted millimetre-scale mechanical properties^24^ but this is, to our knowledge, the first study that shows large-tip spherical indentation alone, without being combined with imaging techniques, does predict some of the millimetre-scale mechanical properties of bone.

### CT porosity

Cortical porosity has been described as one of the most important predictors of bone fragility^13^. Zebaze *et al*. showed that after 80 years old most bone loss is cortical (90%). They found that cortical porosity increases after the age of 50 and worsens exponentially with age^15^. This increased porosity likely contributes significantly to fragility fractures increasing with age^54^. Sundh *et al*. used high-resolution peripheral quantitative CT scans used to estimate porosity at the tibia. In their case-control study they found that tibial cortical porosity was associated with hip fracture cases, independent of BMD measured by DXA^55^. This highlights the importance of porosity. The challenge is achieving the necessary resolution to determine porosity using CT without exposing the patient to high radiation doses. The StrAx1.0 software developed in Melbourne shows some promise in estimating porosity using clinical CT scan images. It automatically selects attenuation profile curves and segments the bone into trabecular, transitional and compact cortical bone. Using this software and low resolution clinical CT (voxel size 740 μm), they found that cortical porosity was associated with non-vertebral fracture risk, independent of bone mineral density^56,57^.

In our study, we used high resolution micro-CT to image the pores in cortical bone. A previous synchrotron CT study that measured cortical porosity^58^ had a similar pixel size to our study (7.5 μm compared to 7 μm in our study). Currey and Shahar classified cortical porosity according to five levels, from large to small: Level 1 is classified as the marrow cavity, level 2 is the level for nutrient arteries, level 3 is the vascular porosity, level 4 is the lacuna-canicular porosity and level 5 is the nanoporosity^59^. Cooper *et al*. stated that from “an imaging perspective, cortical porosity, as a measurable outcome, refers to the vascular porosity”^60^. Carter *et al*. demonstrated that Haversian canals in proximal femoral cortical bone ranged from 29 (+/− 4) to 44 (+/− 9) micrometres in diameter^61^. As a result, most of the vascular porosity of the cortical bone should have been captured in our study.

The study is also unique as it is the first study to measure cortical porosity using micro-CT at the femoral neck and also perform indentation, compression and ultrasound testing on the same samples. Abraham *et al*. previously performed micro-CT at the tibial mid-shaft, as well as performing reference point indentation at the tibia and compared this to mechanical testing of the whole proximal femur. They found that cortical bone at the tibial mid-shaft ranged in porosity from 1.6 to 10.9%, much less porous than the femoral neck cortical bone in our study^16^. Porosity correlated with load to failure (*r* = −0.5) but not as strongly as in our study (*r*_*s*_ = −0.75), which is likely due to the porosity being measured in the same bone being tested, rather than tibial bone in the Abraham *et al*. study^16^. Jenkins *et al*. performed reference point indentation, micro-CT and fracture toughness tests on femoral neck cortical bone. They did not report the individual relationship between CT porosity and bone mechanical properties measured by the fracture toughness tests. They did report the influence of porosity on indentation, though, which is discussed below^62^.

Cortical porosity is only one of the determinants of whole bone mechanical properties. Other factors such as cortical thickness, trabecular microarchitecture, collagen cross-linking, the presence of microcracks and whole bone geometry also contribute to whole bone mechanical properties^63^. Volumetric bone mineral density (vBMD), measured by quantitative CT scanning (using a bone mineral density calibration phantom), has also been shown to correlate well with bone mechanical properties^64^. vBMD is dependent on both the bone mineral content, as well as the porosity of the bone^60,65^. To measure vBMD a density-calibration phantom needs to be within the CT field of view^66^. For this study, the principal aim of performing the CT scans was to resolve the cortical pores so pixel size could not be sacrificed. To also measure vBMD would have meant increasing the field of view and pixel size, compromising the resolution of the pores.

### Spherical indentation

A systematic review in 2017 that assessed indentation studies found no reports of an indentation technique that alone predicted millimetre scale human bone mechanical properties^22^. In rat bone, reference point indentation (RPI) correlated with bone toughness measured by 3-point bending^67^. Further studies on human bone have been published since the 2017 systematic review, though: Jenkins *et al*. showed a significant negative correlation between indentation depth increase (IDI), measured by RPI using the BioDent system (Active Life Scientific, Santa Barbara, USA) and fracture toughness (*r* = −0.4) and derived elastic modulus (*r* = −0.4)^62^. Abraham *et al*. found a significant correlation between IDI using the BioDent reference point indentation system and failure load when compressing the proximal femur (*r* = −0.48). The Abraham *et al*. study therefore indicates a correlation between reference point indentation values and macroscale mechanical properties^16^. Assessing the correlation between spherical depth-sensing indentation and microscale properties, a study by our group published in 2018 found no correlation between indentation moduli using a 1.5 mm diameter spherical indenter tip and compression testing moduli^25^.

The improved correlation in this study could be due to the large-diameter spherical tip used. Jenkins *et al*. demonstrated that indentation results are significantly affected by the porosity of the bone. If an indentation occurs into a pore the results from that indentation are unlikely to be interpretable^62^. In a study by Oyen *et al*.^32^ the issue of indenter tip size and its relation to the pores was discussed. The authors noted that indenting between pores using nanoindentation results in notably different results than indenting at a larger length scale, where the pores are included in the indentation^32^. To solve this problem in a laboratory setting indentation can be guided under microscopy to avoid pores^68^. For translating indentation into a clinical setting, though, microscopy prior to indentation would not be feasible but the size of the indenter tip can be increased to make it larger than the pores. The improvement in correlation with millimetre scale mechanical properties, when compared to the previous study^25^, could be partly due to the larger tip including more of the cortical porosity, being closer in length-scale to the compression testing. It could also be due to improvements in the testing methodology for both the indentation and compression testing when compared to the previous study, though^25^.

Although there was a significant correlation between indentation values and compression testing values, the actual moduli values from indentation testing were low. This is most likely due to the indenter tip being too large to satisfy the boundary conditions for indentation in the small samples. Finite element analysis was performed to test the importance of the sample width when performing indentation using a 6 mm diameter, spherical indenter tip. The bone was modelled as 6 mm in height, the height of the samples in the study, using ANSYS software (ANSYS, Canonsburg, USA). The modelling was performed using half the indenter tip and half the bone to simplify the analysis (Fig. S3, Supplementary Materials 4). Three sample widths were chosen: 3 mm which was equivalent to the dimensions of the samples, 30 mm and 60 mm. The bone was assigned an elastic modulus of 6 GPa to be in the range of values measured by indentation in this study. 10 N of load was applied in the simulation and displacement was calculated. When the sample width was 3 mm, displacement was 9.5 μm. When the sample width was 30 mm and 60 mm the displacement was 7.7 μm. This implies that as the bone sample width is decreased below a critical level the elastic modulus measured by indentation will also decrease. It is, therefore, likely that the lower elastic modulus values recorded in this study are due to the sample widths being small relative to the area of strain underneath the 6 mm indenter tips.

It is noteworthy that when the high porosity sample was excluded from the analysis the trends between indentation and other measurements did not reach statistical significance. We chose to report the results of non-parametric methods of data analysis, which included the higher porosity sample in the analysis, though, because previous studies have reported cortical porosity values of over 40%^15,69^. Although this sample had a higher porosity than other samples in the study, this may just reflect the age group of the patients in this study. The maximum patient age in this study was 81 years old. A study by Rajapakse *et al*. included samples from patients up to the age of 93 years old and found that cortical porosity increased with age. The maximum cortical porosity in their study was 50%, as measured by micro-CT^69^. McCalden *et al*. also showed that cortical porosity increases with age^70^. Thus, we did not feel it was right to exclude the sample from the analysis due to it being more porous than the other samples as some older patients having hip surgery may have cortical porosity values in this range.

### Resonant ultrasound spectroscopy

This is the first study to report using resonant ultrasound spectroscopy (RUS) to measure the elastic properties of femoral neck cortical bone. RUS has been used to measure cortical bone from the femoral mid-shaft and tibia previously^48,71^. RUS was able to measure the full stiffness tensor of 19 of the 20 cortical bone samples in this study. One sample had a very high porosity (40%) and CT imaging indicated that there was some trabecular bone in the sample. The patient had a thin cortex and even though, when cutting the sample, macroscopically it appeared to be cortical bone, some of this cortical bone had possibly been remodelled to “trabecularised cortical bone” by endocortical resorption and coalescence of the cortical pores, as described by Zebaze and Seeman^13^. For this reason, RUS was not performed on this sample as the RUS model has only been developed for a homogenous material at the millimetre scale. Research is currently being carried out into using RUS to measure trabecular bone^72^. The RUS technique has not been previously used for cortical bone from the femoral neck. This study provides the first comparison between cortical bone apparent elastic modulus determined from RUS and mechanical parameters obtained from a quasi-static mechanical test. The ultimate compressive strength was found to be correlated to RUS E3. However, no correlation was observed between the RUS E3 moduli and compression testing elastic moduli (Table 2). This may in part be due to measurement errors. Although LVDTs were used to improve the measurement precision, it is possible that techniques such as high-resolution video extensometry and other methods that reduce end-effects could have further improved strain measurement^44,50^. It is worth noting that the overall consistency of the ultrasound measurements is evidenced through the correlation between BWUS C33 and RUS E3. A very high correlation is not expected between these two quantities because in theory, the stiffness coefficient C33 is a function of E3 and other elastic coefficients such as the Poisson’s ratios.

The average elastic moduli measured by compression testing were substantially smaller than the average RUS E3 moduli (Table 1). Other studies have previously reported ultrasound testing resulting in higher moduli than compression testing^45,73^. Rho *et al*. account for this difference by explaining that ultrasound testing is equivalent to testing at a higher strain rate^45^. Bone is viscoelastic, meaning that its stiffness properties vary depending on the rate at which it is deformed, and at high strain rates bone has been shown to have a higher elastic modulus^74^. Han *et al*. demonstrated that ultrasound velocity measurements of bone elastic moduli correlated better with elastic moduli measured by mechanical testing at a higher strain rate than elastic moduli measured by mechanical testing at a quasistatic strain rate^75^.

### Limitations

This study primarily investigated bone stiffness. It would be useful to expand this study to investigate fracture toughness also. Although ultimate compressive strength (UCS) measurements were made in this study, the fracture toughness of bone does not necessarily correlate with its strength^52^. Twenty samples from twenty patients were used in this study. To assess the strength of the correlations further, this number could be expanded. For the CT scans, it would have been useful to have a bone density calibration phantom in the scanner. This was considered at the start of the study. However, the principal aim of CT scanning was to measure the porosity and to achieve this CT resolution a small field of view was needed. Expanding the field of view to include a phantom would have led to an increased pixel size and potentially missing some of the cortical pores. The volumetric bone mineral density (vBMD) of cortical bone, as measured by quantitative CT scanning with the use of a density calibration phantom (pixel size 97 μm), has been shown to correlate strongly with cortical porosity (*r* = −0.86)^65^. It would have been useful to perform quantitative CT scanning of the samples to determine the vBMD as quantitative CT is currently used in clinical practice. In future studies assessing multiple bone mechanical property measurement modalities, quantitative CT should be included as a measurement tool. Finally, it is worth noting that the bone used in this study came from the femoral necks of patients undergoing hip replacement surgery for osteoarthritis and caution is, therefore, advised if the findings from this study are extrapolated to the population at large.

### Measuring cortical porosity: future research directions

Measuring cortical porosity without exposing a patient to large amounts of ionising radiation is an ongoing challenge and suggested goal for future studies. We suggest three avenues for future research: A CT technique, a Magnetic Resonance Imaging (MRI) technique and an ultrasound technique.

The StrAx1.0 software, developed in Melbourne^76^, is one possible solution for measuring cortical bone porosity without a large radiation exposure. The technique does not directly measure cortical porosity; Clinical CT scans are used and the software automatically selects attenuation profiles and segments the images into cortical, transitional and trabecular bone^56^. In one study, high resolution peripheral quantitative CT (HR-pQCT) scans (voxel size 82 *μ*m) were performed on cadaveric bones, as well as micro-CT scans (19 *μ*m voxel size). The cortical porosity calculated by the StrAx1.0 software using the HR-pQCT scan images compared very well with the cortical porosity calculated by the micro-CT images (r^2^ = 0.87)^76^. HR-pQCT scans can be performed on patients’ wrists *in vivo*. The StrAx1.0 software, thus, enables a doctor or researcher to reasonably calculate a patient’s cortical bone porosity at the distal radius. The StrAx1.0 software has also been applied to normal clinical CT scans (voxel size 740 *μ*m) to measure cortical porosity. Cortical porosity measured with this technique was associated with patients fracturing their bones, independent of bone mineral density (BMD) and Fracture Risk Assessment (FRAX) score^56^.

Another potential solution for measuring porosity *in vivo* is magnetic resonance imaging (MRI) technology. Using fast spin-echo sequences (FSE) and high resolution (3-Tesla scanner), cortical porosity can be measured from the water content in the bone. In one study by Bae *et al*.^77^, FSE MRI scans and micro-CT scans were used to measure cortical porosity in cadaveric tibial bone. There was a high correlation (*r*^2^ = 0.83) between porosity measured by micro-CT and porosity measured by FSE MRI^77^. This technique could potentially be developed and made available in clinical practice, once validated *in vivo*.

Finally, a guided-wave ultrasound technique has been developed for measuring cortical porosity and thickness^78^. This technique calculates cortical porosity and thickness by recording the guided wave Lamb modes and uses computer modelling to calculate the porosity and cortical thickness from these modes^78,79^. In a cadaveric study, this ultrasound technique and micro-CT scanning were used to calculate cortical porosity and cortical thickness. The agreement between the ultrasound and micro-CT techniques for cortical porosity was *r*^2^ = 0.63 and, for cortical thickness, *r*^2^ was 0.89. This technique is currently being investigated in a clinical trial.

## Conclusions

Cortical bone porosity, as measured by micro-CT, correlated with the millimetre-scale apparent elastic modulus and ultimate compressive strength of femoral neck cortical bone. A correlation was also observed between spherical indentation moduli and bone mechanical properties at the millimetre scale, an improvement on previous indentation studies in the literature using smaller indenter tips. As cortical bone porosity reflects millimetre-scale bone mechanical properties at the femoral neck we would recommend further research into technologies that can safely measure cortical porosity *in vivo*.

## Materials and Methods

### Sample preparation and summary of the testing methods

Femoral heads and necks were retrieved from twenty patients who underwent elective total hip replacement for osteoarthritis (12 female, 8 male, mean age 66 years, age range: 48–81 years). All patients gave written, informed consent for the use of their tissue for research and ethical approval for this study was received (Imperial Tissue Bank number R13004a, Wales Research Ethics Committee number 12/WA/0196). The study was carried out in accordance with the relevant guidelines and regulations from Imperial Tissue Bank.

**Figure 8:**
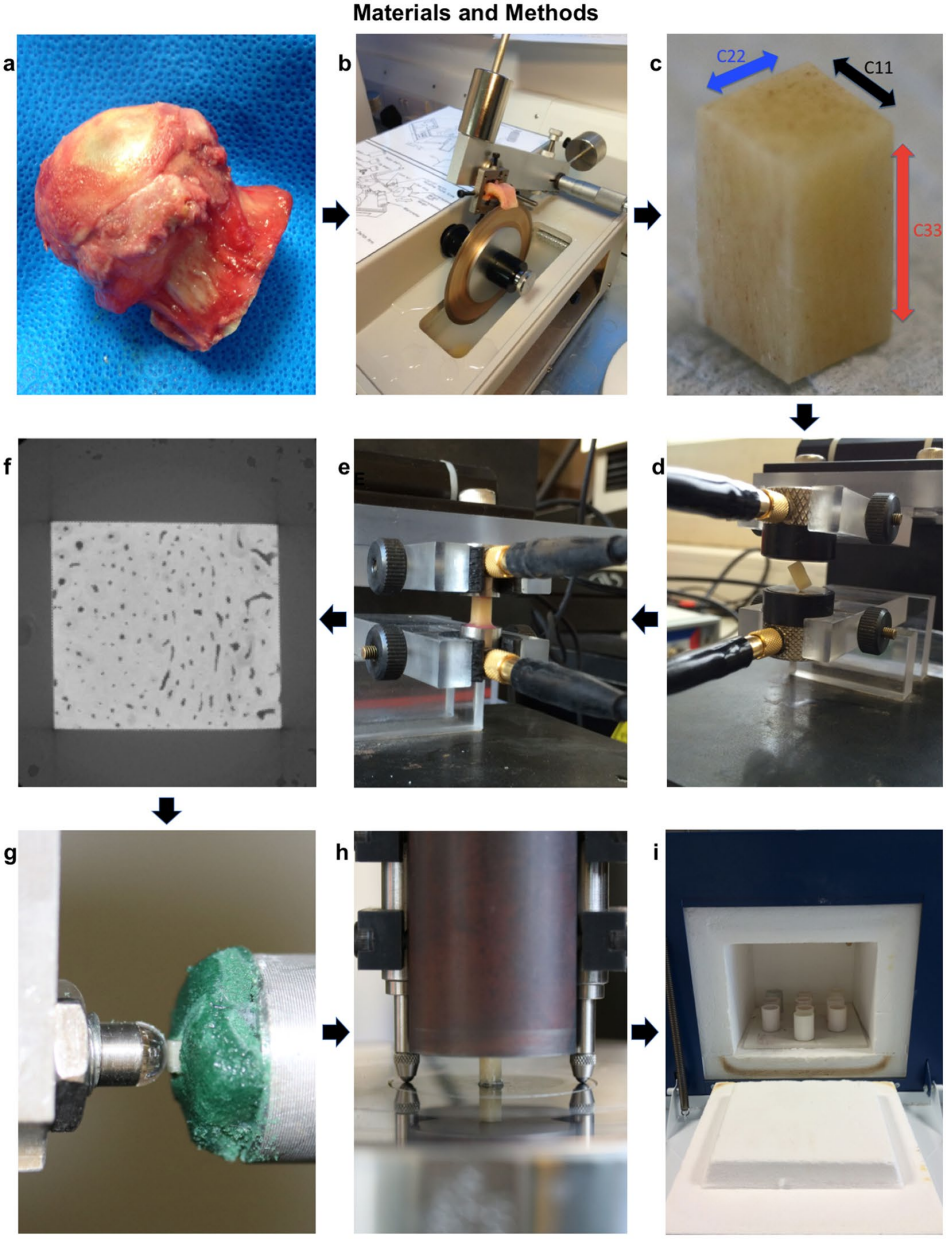
Materials and Methods. (**a**) Femoral head and neck collected from hip replacement section. (**b**) Small section of bone cut from the medial femoral neck cortical bone (calcar region) using a diamond wafering blade and custom clamp. (**c**) Image of a prepared sample showing a 3 × 3 × 6 mm, rectangular, parallelepiped, cortical bone section. The longitudinal axis (axis 3, corresponding to the C_33_ stiffness tensor) is aligned with the osteons. (**d)** Sample held on two opposite corners between two ultrasound transducers for resonant ultrasound spectroscopy. (**e**) Sample standing between two ultrasound transducers for ultrasound bulk wave velocity measurements in the C_33_ direction. (**f**) Axial section (3 mm × 3 mm) from micro-computed tomography imaging, displaying the porosity of the cortical bone. (**g)** Sample undergoing spherical indentation testing in the axis 3 direction. The bone is surrounded with floral foam which is kept wet throughout testing by irrigation with phosphate-buffered saline. (**h)** Compression testing for measuring the compression apparent elastic modulus and the ultimate compressive strength in the axis 3 direction. The bone is placed unconstrained between two platens and compression testing is performed using a dual-axis Instron materials testing machine. Strain is measured by two linear variable differential transformers (LVDTs), either side of the bone. (**i**) Bone samples for ashing, contained in ceramic crucibles within a furnace.

Sample preparation was carried out according to a previously published protocol^25^. Samples (Fig. 8a) were frozen after surgery and thawed fully before testing in 0.9% saline solution. A diamond wafering blade saw (Isomet, Buehler, Germany) and custom-made, additively-manufactured clamp (Fig. 8b) were used to cut 3 × 3 × 6 mm rectangular parallelepiped cortical bone samples (Fig. 8c) from the thickest part of the medial calcar region of each femoral neck. Samples were 3 mm in width in the radial direction (axis 1), 3 mm in the transverse direction (axis 2) and 6 mm in the longitudinal direction (axis 3) and were cut such that axis 3 (6 mm in length) was parallel with the direction of the osteons. Samples were then measured and tested using the following summary workflow, displayed in Fig. 8. Mass density was measured, followed by resonant ultrasound spectroscopy and ultrasound bulk wave velocity measurements, performed to determine the elastic modulus in the axis 3 direction. High-resolution micro-CT scanning was then performed to determine porosity. Following this, the samples underwent spherical indentation in the direction of axis 3 and the elastic modulus was determined. The samples then underwent compression testing to failure in the axis 3 direction and the compression apparent elastic modulus (CAEM) and ultimate compressive strength (UCS) were calculated.

### Mass density measurements

Sample dimensions were measured with digital callipers (precision ± 0.05 mm). Four measurements were performed and the mean was calculated and used for the volume calculation. Measurements were taken by using the callipers at different locations to account for possible parallelism defects. Samples were weighed in their fully hydrated state immediately before and immediately after the resonant ultrasound spectroscopy (RUS) tests. Four measurements were made before and four after RUS testing using scales with a precision of 0.1 mg. The mean of these eight measurements was taken as the sample wet mass, which was used for the density calculation, together with the volume (mass divided by volume).

### Resonant ultrasound spectroscopy

Resonant ultrasound spectroscopy (RUS) was performed according to the method by Bernard *et al*.^48,80^ and calculated the orthotropic stiffness tensor of the cortical bone samples. Thawed samples were tested after being immersed in phosphate buffered saline for fifteen minutes to ensure they were fully hydrated. Samples were positioned and held between two ultrasound transducers (V154RM, Panametrics, Waltham, MA) on opposite corners, to be as close as possible to stress-free boundary conditions (Fig. 8d). Samples were excited with an ultrasound pulse and the frequency responses of the samples were recorded using a vectorial network analyser (Bode 100, Omicron electronics GmbH, Austria). RUS is a method that involves matching experimental resonant frequency measurements to model-predicted frequencies, as described in detail in Bernard *et al*.^48^. The model assumes orthotropic symmetry of the bone sample. Briefly, 6 measurements were performed on each sample with the sample rotated by approximately 15 degrees in between each measurement, which allowed the six measurements to be combined and more frequency peaks to be detected. Indeed, bone is a high damping material so one measurement is not always sufficient for detecting enough frequency peaks^48^. The orthotropic stiffness constants Cij (ij = 11, 22, 33, 12, 13, 23, 44, 55 and 66) (Voigt notation) were automatically calculated by optimizing the misfit function between the experimental and model predicted resonant frequencies (inverse problem), which was formulated in a Bayesian framework^80^. The prior information on the distribution of the stiffness constants, required for the Bayesian analysis, was taken from a previous study on human femoral cortical bone^14^. In this study, the differences between experimental and predicted frequencies were less than 1%. Assuming orthotropic symmetry, the complete set of stiffness constants (and the inverse elasticity matrix in terms of engineering moduli) were calculated. The elastic modulus in the E3 principal direction was used for comparison with other methods (Fig. 8c).

### Bulk-Wave ultrasound velocity (BWUS) measurements

Longitudinal stiffness coefficients (*c*_*ii*_) were calculated from the BWUS velocity (*v*_*ii*_) and the apparent density (*ρ*) as,

1 $${c}_{ii}=\rho {v}_{ii}^{2}$$

where *i* is 1, 2 or 3, and denotes the propagation direction of the longitudinal wave^81^.

Ultrasound measurements were performed using a pair of longitudinal transducers of 5 MHz central frequency (V110RM Panametrics) in contact with the sample surface (Fig. 8e). Thawed samples were tested after being immersed in phosphate buffered saline for fifteen minutes to ensure they were fully hydrated. A pipette-drop of distilled water was used to improve the contact with the bone. An ultrasound pulse was generated by a pulser (200 MHz, 3 dB, ultrasound bandwidth, Panametrics 5900PR (Olympus, Japan)), emitted by one transducer and received by the other. The received signal was digitised and stored using an acquisition card (Acqiris DP240, Acqiris, Switzerland) and post-processed in MATLAB (Mathworks, USA). The bulk wave velocity was calculated by dividing the sample length by the time of flight. The time of flight was recorded as the time from the ultrasound pulse being emitted to the signal’s first deviation from zero, as explained in more detail in Peralta *et al*.^81^. Six successive measurements with repositioning of the samples in between were done for all measurements and the average of the six velocity measurements was used to calculate the stiffness. Bulk wave velocity ultrasound measurements were performed in addition to RUS because bulk wave velocity measurements of bone stiffness have been more frequently reported in the bone literature.

### Micro-CT scanning for determining porosity

Samples were placed in custom-made, additively-manufactured, cylindrical, polyamide-12 containers. The containers were 30 mm tall and had an inner diameter of 5 mm (designed to be just wide enough to fit the samples). Samples were placed at the bottom of the container and then immersed in phosphate buffered saline. This was to ensure the samples did not dry out during CT scanning. Samples were scanned individually in a high-resolution micro-CT scanner to a pixel size of 7 micrometres (μm), to determine cortical porosity (Zeiss Xradia 510 Versa, 50 kV, 4 W, 0.4x objective, 8 second exposure, Bin 2, LE1 filter, 1601 projections over 360 degrees rotation, with a total scan time of 7 hours). Images (Fig. 8f) were reconstructed using the scanner software, cropped using ImageJ (ImageJ, NIH, USA) and then exported as 8 bit data to Avizo (ThermoFisher Scientific, Oregon, USA) for visualisation and quantitative analysis. Thresholding by Hounsfield units was used to separate the pore volume from the bone by image segmentation in Avizo. The porosity was defined as the total pore volume divided by the total sample volume.

### Spherical indentation

Samples underwent indentation in the axis 3 direction using a 6 mm diameter, sapphire indenter tip, mounted in a NanoTest 3 (Micro Materials Ltd., Wrexham, UK), a depth-sensing micro-indentation machine. The apparent elastic modulus was calculated using the unloading curve following indentation, using the Oliver-Pharr method^33,82^. 16 indentations were performed per sample, 200 micrometres apart, on a pre-defined indentation grid. The mean value of the central four indentations was used for subsequent analysis as the more peripheral indentations were judged to be too close to the sample edges, leading to less reliable measurements. Samples were adhered to the sample holder using cyanoacrylate glue (Fig. 8g). Floral foam was wrapped around the bone sample below the indentation surface. The foam was kept moist by applying phosphate buffered saline to the foam by pipette every ten minutes. In this way, the samples were kept moist during indentation. A preload of 200 micro-Newtons was used to engage the indenter tip with the bone surface. Load was then applied at a loading rate of 0.1 Newtons per second (*N*/*s*) up to a maximum load of 10 *N* followed by 60 seconds holding at this load, before fully unloading at a rate of 0.3 *N/s*. Data were corrected for machine compliance by a calibration test using a Berkovich tip and tungsten sample. The apparent elastic modulus for each indentation was calculated from the load-displacement data, using the Oliver-Pharr technique, using software inbuilt into the NanoTest 3 indentation machine (Micro Materials Ltd., Wrexham, UK). Full details of the method are in the original paper by Oliver and Pharr in^33^ and the subsequent paper, which included spherical tip indentation, published in^82^. Briefly, load (*P*) and displacement (*h*) are recorded by the indentation machine. From the load-displacement graph, three values are recorded: the maximum displacement (*h*_max_), maximum load (*P*_max_) and the slope of the upper portion of the unloading curve (d*P*/d*h*), which is the stiffness, *S*. To determine the upper portion of the unloading curve a power law fit is applied, with the formula below, where α and *m* are fitting constants:

2 $$P=\alpha {(h-{h}_{f})}^{m}$$

Following this, the contact depth, *h*_*c*_, is calculated, where *h*_*f*_ was determined from the power law fit:

3 $${h}_{c}=\frac{{h}_{\max }+{h}_{f}}{2}$$

The indentation area, *A*, is then calculated by:

4 $$A=2\pi R{h}_{c}$$

where *R* is the indentation radius, which was 3 mm in this study. After this, the reduced modulus is calculated:

5 $${E}_{r}=\frac{\sqrt{\pi }}{2}\times \frac{S}{\sqrt{A}}$$

The elastic modulus, *E*, is then calculated from the below formula, where *v* is the Poisson’s ratio and *i* is the indenter tip material. Bone was assigned a Poisson’s ratio of 0.33^83^, and sapphire was assigned an elastic modulus of 420 GigaPascals (GPa) and Poisson’s ratio of 0.24^84^.

6 $$\frac{1}{{E}_{r}}=\frac{(1-{v}^{2})}{E}+\frac{(1-{v}_{i}^{2})}{{E}_{i}}$$

### Compression testing

Samples were placed unconstrained between two polished platens in a dual-axis, servohydraulic, materials testing machine (Instron 8874, Instron Ltd., High Wycombe, U.K.). Displacement was measured by two linear variable differential transformers (RDP D6/05000A, RDP Electronics Ltd, UK) either side of the bone and the average of the two readings was used (Fig. 8h). Load was applied under displacement control in the axis 3 direction of the bone with an initial relative ramp to 0.01 mm at a displacement rate of 1.8 mm/minute followed by 10 preconditioning cycles at an amplitude of 0.01 mm and a frequency of 0.5 Hz. These preconditioning cycles were carried out to ensure that there was good contact between the bone surfaces and the platens^44^, and to minimise end-effects^85^. Low strain levels were used in the preconditioning cycles to minimise the risk of plastic deformation to the bone samples, as advised by Linde *et al*.^86^, Keaveny *et al*.^85^ and Zhao *et al*.^50^. Following preconditioning, samples were loaded to failure with a relative ramp to 2 mm displacement at a rate of 1.8 mm/minute. This corresponds to a strain rate of 0.005/s, considered to be the quasi-static strain rate of bone from previous studies^44,74,87^. The load-displacement data and sample dimensions were input into a custom MATLAB (Mathworks, USA) script that first converted the data into stress and strain and then plotted the stress-strain curve. To calculate the elastic modulus, the script calculated the steepness of multiple, best-fit straight lines over 0.2% strain ranges, plotted onto the loading curve, with varying origins. The maximum steepness line was used to calculate the apparent elastic modulus, ensuring the maximum modulus was calculated, similar to the method by Keaveny *et al*.^44^. In addition, the maximum point on the stress-strain curve was recorded as the ultimate compressive strength.

### Ashing to determine bone mineral content

Samples were placed individually in ceramic crucibles and placed in a furnace (Fig. 8i). 10 crucibles could be positioned into the furnace (Lenton EF 11/8B, Lenton, UK) at one time so samples were ashed in two batches using the method reported by Wang *et al*.^88^. Bone samples were first dried at 70° Celsius overnight in the furnace. The furnace was then heated to 800° Celsius and samples were heated at this temperature for 3 hours. The ashed samples were then weighed and this was recorded as the ash mass. The bone mineral density by ashing (BMDA) was calculated by dividing the ash mass by the sample volume.

### Statistical analysis

The relationships between all the measurements were assessed by ordinary least squares regression and the Pearson’s correlation coefficient, *r*, together with *p* values, were calculated using IBM SPSS v24 (IBM, USA). For data that were not normally distributed, the correlations between measurements were assessed by Spearman’s Rank Correlation Coefficient, *r*_*s*_, with *p* values, using IBM SPSS v24 (IBM, USA). Graphs were plotted using the software Origin (OriginLab, USA). Correlation coefficients were reported as strong if greater than 0.6, moderate if between 0.4 and 0.6, and weak if less than 0.4^89^ Multiple linear regression analyses were also performed for normally distributed data using IBM SPSS v24.

## Supplementary information

Supplementary Materials 1

Supplementary Materials 2

Supplementary Materials 3

Supplementary Materials 4

## Data Availability

All data generated or analysed during this study are included in this published article and its Supplementary Information files. Additional datasets generated during the current study are available from the corresponding author on reasonable request.
