# Supplementary material for: Computed tomography porosity and spherical indentation for determining cortical bone millimetre-scale mechanical properties
Source: Sci Rep. 2019 May 15;9:7416. doi: 10.1038/s41598-019-43686-6 (PMC6520408; doi:10.1038/s41598-019-43686-6)
Supplement: Supplementary file 4 — Supplementary Materials 4 [file 41598_2019_43686_MOESM4_ESM.pdf]

Supplementary Materials 4

Title of the manuscript: Computed tomography porosity and spherical indentation for determining cortical bone millimetre-scale mechanical properties

Authors: Oliver R Boughton[1,2*], Shaocheng Ma[1,2], Xiran Cai[3], Liye Yan[4], Laura Peralta[3], Pascal Laugier[3], James Marrow[4], Finn Giuliani[5], Ulrich Hansen[2], Richard L Abel[1], Quentin Grimal[3], Justin P Cobb[1]

Affiliations

[1]The MSk Lab, Department of Surgery and Cancer, Imperial College London, United Kingdom;

[2]The Biomechanics Group, Department of Mechanical Engineering, Imperial College London, United Kingdom;

[3]Sorbonne Université, INSERM, CNRS, Laboratoire d'Imagerie Biomédicale, F-75006 Paris, France;

[4]Department of Materials, University of Oxford, United Kingdom;

[5]Centre for Advanced Structural Ceramics, Department of Materials, Imperial College London, United Kingdom.

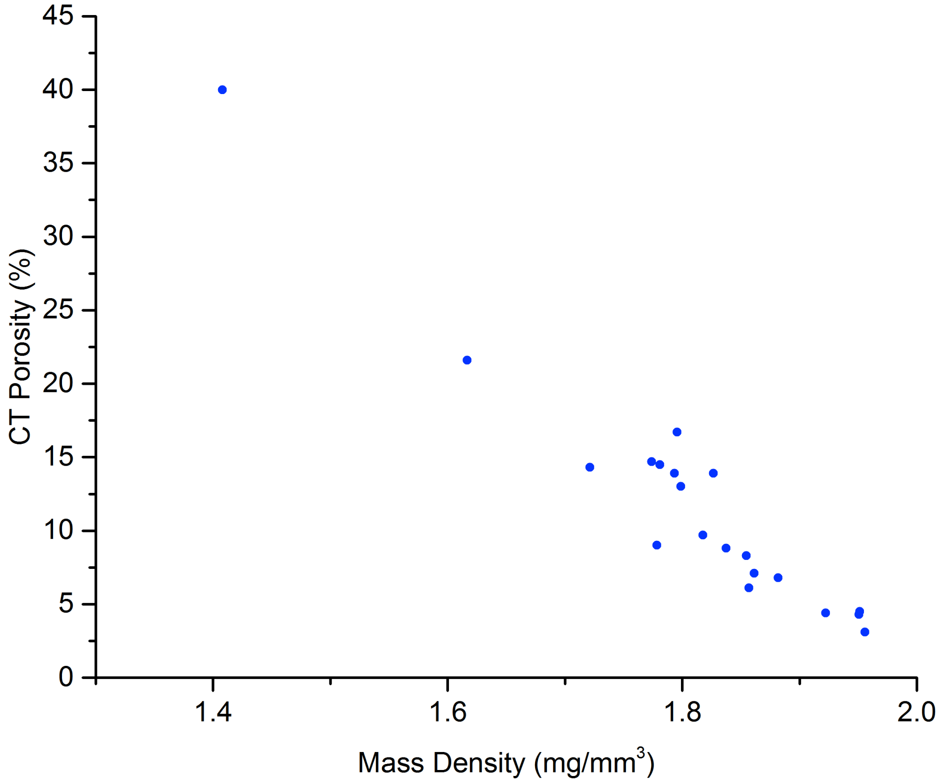

Figure S1. Scatter plot comparing the CT porosity and mass density measurements. The Spearman's Rank Correlation Coefficient, r_s, was -0.93, $p < 0.001$.

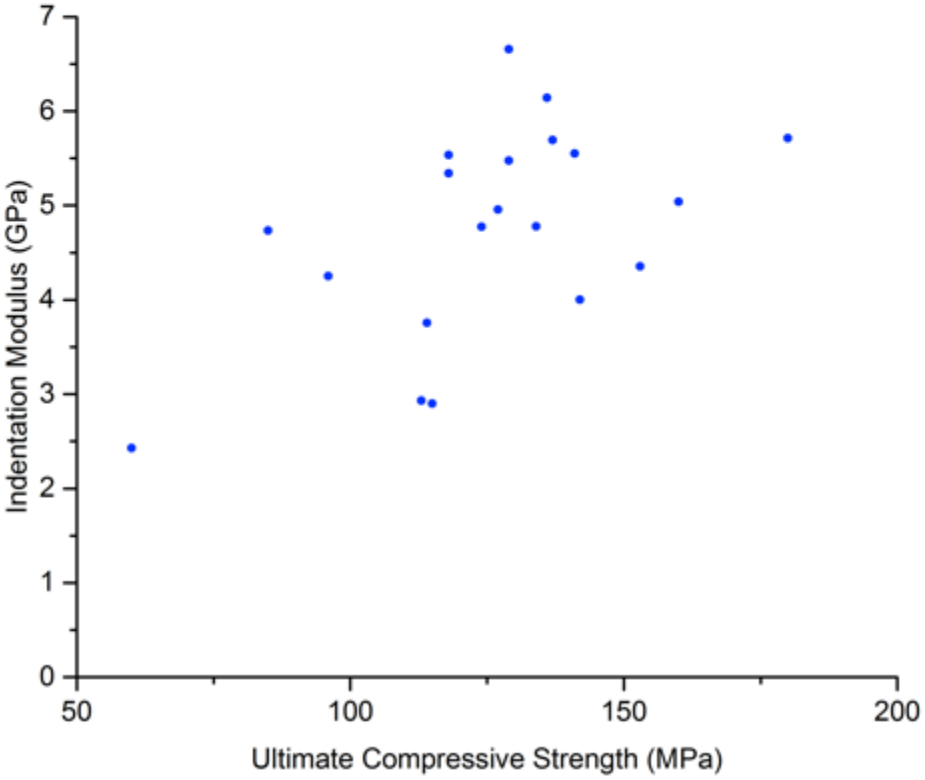

Figure S2. Scatter plot comparing the indentation modulus values with the ultimate compressive strength results. The Spearman's Rank Correlation Coefficient, r_s, was 0.48, $p = 0.03$.

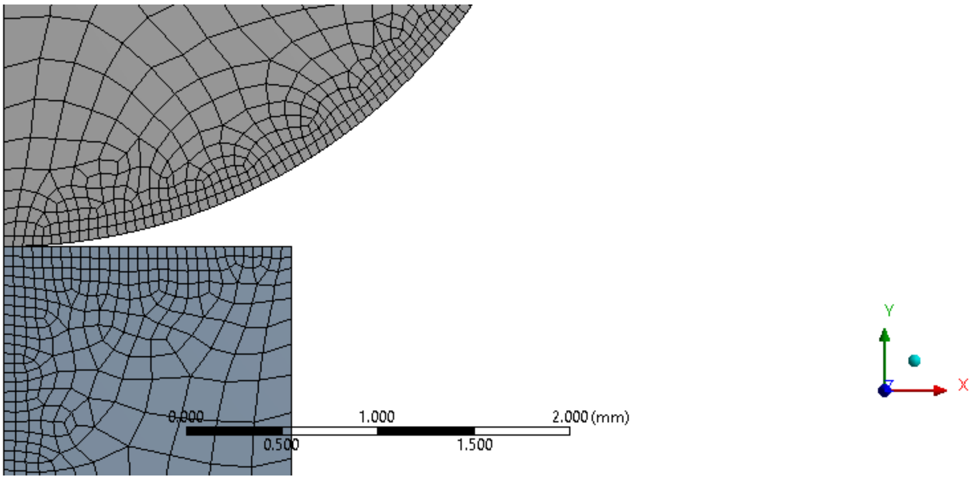

Figure S3: Finite element analysis of spherical indentation using ANSYS (ANSYS, Canonsburg, USA). The spherical indenter tip is modelled in grey, with the bone specimen in blue. The bone specimen width was varied (3, 30 and 60 mm).
